# Difference in Eye Gaze for Floor Apportionment in Native- and Second-Language Conversations

**DOI:** 10.1007/s10919-017-0262-3

**Published:** 2017-09-09

**Authors:** Koki Ijuin, Ichiro Umata, Tsuneo Kato, Seiichi Yamamoto

**Affiliations:** 1 Graduate School of Science and Engineering, Doshisha University, 1-3 Tatara, Miyakodani, Kyotanabe, Kyoto 610-0321 Japan; 2 Faculty of Science and Engineering, Doshisha University, Kyotanabe, Kyoto Japan; 3 Present Address: KDDI Research, Inc., Fujimino, Saitama Japan

**Keywords:** Eye gaze, Multiparty conversation, Floor apportionment, Second-language conversations

## Abstract

In face-to-face communication, eye gaze is known to play various roles such as managing the attention of interlocutors, expressing intimacy, exercising social control, highlighting particular speech content, and coordinating floor apportionment. For second language (L2) communication, one’s perception of eye gaze is expected to have more importance than for native language (L1) because eye gaze is assumed to partially compensate for the deficiencies of verbal expressions. This paper examines and clarifies the efficiency of the function of eye gaze in the coordination of floor apportionment through quantitative analyses of eye gaze during three-party conversations in L1 and L2. Specifically, the authors conducted ANOVA tests on the eye-gaze statistics of a speaker and two listeners during utterances while focusing on whether floor-switch occurs subsequent to the utterance. The analysis results show that the listener who is gazed at more by the speaker is more likely to be the next speaker with a higher probability in L2 than in L1 conversations. Meanwhile, the listeners gaze more at the speaker in L2 than in L1 conversation for both the utterances just before a floor switch and cases with no floor switch. These results support the observation that the eye gaze of the speaker is efficient for floor apportionment in L2 conversations and suggest that longer listeners’ eye gazes in L2 conversations also function efficiently in smooth floor apportionment.

## Introduction

In typical human–human face-to-face interactions, the interlocutors use not only speech and language but also a wide variety of paralinguistic means and nonverbal behaviors to signal their speaking intentions to the partner (Kendon 1967), to express intimacy (Mehrabian and Wiener 1967; Mehrabian and Ferris 1967), and to coordinate their conversation (Clark 1996). Gaze is one of the strongest and most extensively studied visual cues in face-to-face interaction, and it has been associated with a variety of functions, such as managing the attention of interlocutors (Vertegaal et al. 2001), expressing intimacy and exercising social control, highlighting the information structure of the propositional content of speech, and coordinating turn-taking (Duncan 1972; Kendon 1967).

The fundamental gaze patterns related to turn negotiation were discussed in Kendon (1967), who demonstrated that speakers look away at a turn’s beginning and look back to their partners near the turn’s ending. Kendon (1967) suggested that eye gaze activities such as gazing at or avoiding conversational partners might be used for some functions of turn organization during two-person conversations. He stated there were at least four such functions: (1) to provide visual feedback; (2) to organize the conversation’s flow; (3) to interpret emotions and relationships; and (4) to concentrate on understanding the utterance by shutting out visual information. Argyle and Cook (1976) also found that participants gaze nearly twice as much while listening as they do while speaking. In contrast, Beattie (1978) reported that there was no relation between eye gaze and floor apportionment under the experimental condition in which two participants play different social positions. Those results suggest that eye gaze activity combines many functions and that the condition of conversational setup might change the relative importance of these functions (Kleinke 1986).

Those studies mainly dealt with two-party dialogues, not multiparty conversations where the features used for managing turn control may be different from those used in two-party dialogues. In such multiparty conversations as a group of people informally chatting with each other or people attending a more formal meeting, it is obvious that the coordination and interaction cannot be managed in a similar way to how it is done in dialogues between two speakers who share the responsibility for coordination. As for triad conversations, Kalma (1992) reported that the recipient of prolonged gaze, i.e., the participant who is gazed at by the speaker during the silence after his/her utterance, tended to take the floor. Leaner (2003) also reported that the speaker anticipates the next speaker explicitly in many ways, including eye gaze. For turn management with eye gaze, the speaker signals the assumed next speaker by their gaze, thus requiring the gazed-at participant to see that gaze while the other participant also grasps the expectation that someone else will speak next. These studies showed that the eye gaze of the speaker in the conversations has a relation to floor apportionment in multiparty conversations.

Related studies have been presented within several research communities, including human–computer interaction, machine learning, speech processing, and computer vision, with the aim of furthering our understanding of human–human communication and multimodal signaling of social interactions (Gatica-Perez 2009; Pentland 2005; Vinciarelli et al. 2009). In these research areas, Vertegaal et al. (2001) discussed the importance of gaze in multiparty conversations for signaling conversational attention, and Jokinen et al. (2013) showed that the speaker’s gaze is important for coordinating turn taking in multiparty conversations and that partners pay attention to the speaker’s gaze behavior.

These findings on human–human interactions were mainly obtained from conversations held in the mother tongue (L1), and little is known of the effect of linguistic proficiency on multimodal conversations. Second-language (L2) conversations are commonly observed in daily life with the current wave of rapid globalization, and the proficiency of conversational participants typically ranges widely from low to high. Such differences in proficiency can cause serious miscommunication and may disrupt collaboration by both native and non-native speakers in human–human communication (Beyene et al. 2009). Uneven proficiency in L2 may also lead to uneven opportunities for participation in conversations. A multiparty conversation consists of “ratified participants” (Goffman 1976), while participants with poorer proficiency might be relegated to “side participant” status regardless of their level of proficiency in the tasks they are working on collaboratively. Considering the current wave of rapid globalization, it has become an essential task to correctly understand human–human interaction in L2 conversations.

As for eye gaze in L2 conversations, which was expected to have almost the same functionality as it has in L1 conversations, Hosoda (2006) suggested that language proficiency may affect the functions that eye gaze performs, and Veinott et al. (1999) found that non-native speaker pairs benefited from using video communication in route-guiding tasks, whereas native speaker pairs did not. They argued that this was because video transmitting facial information and gestures helped the non-native pairs to negotiate a common ground, whereas this did not provide significant help for the native pairs. These observations suggest that eye gaze and visual information play more important roles in establishing mutual understanding in L2 conversations from those in L1 conversations.

To quantitatively and precisely analyze the difference in eye gaze between L1 and L2 conversations, Yamamoto et al. (2015) created a multimodal corpus of three-party conversations for two different conversation topics in L1 and L2. In this way, it was possible to compare the features of utterance, eye gaze, and body posture in L1 and L2 conversations conducted by the same interlocutors (Yamamoto et al. 2015). To compare the features of eye gaze in L1 and L2 conversations, they used two metrics: (1) how long the speaker was gazed at by other participants during her or his utterance (listener’s gazing ratio) and (2) how long the speaker gazed at other participants during her or his utterance (speaker’s gazing ratio). The experimental results show that the averages of speaker’s gazing ratios are almost the same in four kinds of conversations (two different conversation topics and two different conversation languages), whereas the averages of listener’s gazing ratios are larger in L2 conversations than in L1 conversations for both conversation topics. Ijuin et al. (2015) classified three interlocutors into current speaker, next speaker, and other participant (not next speaker) by considering the transition of speaker in the conversation and, furthermore, compared the speaker’s gaze activities in L1 and L2 from the perspective of conversational interaction. The analysis revealed two observations: (1) the speaker gazes at the interlocutor who is to be the next speaker more in L2 than in L1 conversations, whereas the averages of speakers’ gazing ratios are almost the same in both L1 and L2 conversations; (2) not only the next speaker but also the other participant, who is not gazed at so much by the current speaker, gazes at the current speaker more in L2 conversations.

These results suggest that the function of eye gaze in multiparty conversations in L2 is more important in coordinating floor switch than those in L1, probably to compensate for the lower communicative competence in L2 conversations. There is a possibility that the speaker proactively shows who is to be the next speaker by his/her eye gaze, and the listeners need to capture the speaker’s communicative signals so as to be ready to find an opportunity to take the floor. There is another possibility that the speaker monitors the next speaker by grasping the communicative signals from the partner who is about to take the floor.

In this study, we classify utterances into ones just before floor switch and the others, and then conduct detailed analyses of the eye gaze activities during both classes of utterances in L1 and L2 conversations to test the above hypothesis. We aim to answer two specific questions in this study:

- Does the speaker’s eye gaze affect floor apportionment more significantly in L2 conversations than in L1 conversations?
- Do longer listeners’ eye gazes in L2 conversations function efficiently in smooth floor apportionment?

This paper is structured as follows. We introduce the multimodal corpus we used in Sect. 2, show the methodology in Sect. 3, and present analysis results for the relation between eye gaze and floor apportionment in Sect. 4. Then, we discuss those results in Sect. 5 and conclude with a summary in Sect. 6.

## Multimodal Corpus

**Fig. 1:**
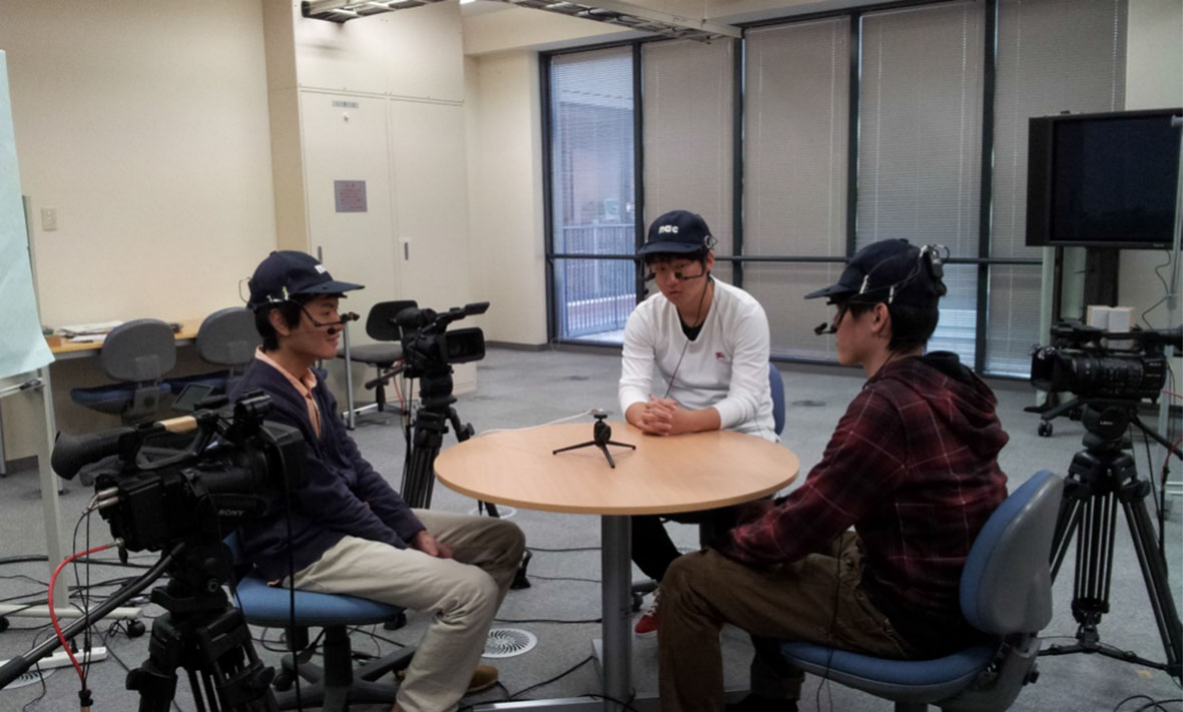
Experiment setup

A multimodal corpus created by Yamamoto et al. (2015) was used for our analyses of eye gaze for coordinating floor apportionment. The data of this multimodal corpus were collected from triad conversations in Japanese as the interlocutors’ native tongue and in English as their second language. Three subjects participated in a conversational group, sitting in a triangular formation around a table as shown in Fig. 1. Three SONY video cameras were used, each capturing the face and upper body of one participant, and three NAC EMR-9 eye trackers were used to record eye gaze.

The multimodal corpus included input from a total of 60 subjects (23 females and 37 males: 20 groups). The corpus contains L1 and L2 conversations held in two types of conversation. The first type was free-flowing, which was natural chatting that covered various topics such as hobbies, weekend plans, studies, and travel. The second type was goal-oriented, in which they collaboratively decided on issues related to a specific topic such as what to take with them on trips to uninhabited islands or mountains. Each conversation was carried out for approximately 6 min.

**Fig. 2:**
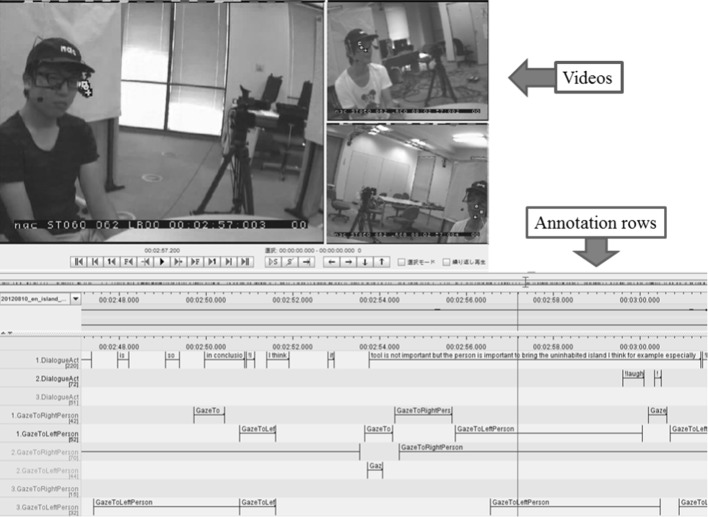
Example of annotation results using ELAN. The *upper side* shows videos recorded by the eye trackers for each participant, and the *lower side* shows rows of annotation items

The multimodal corpus was manually annotated in terms of the time spans for utterances, backchannel, laughing, and eye movements. Each utterance was segmented from speech at inserted pauses of more than 500 ms, and its annotation was composed of the start and end times and the attributes of utterance. The annotation of gaze events was also composed of the start and end times and attributes such as “gaze at the right-side person”, “gaze at the left-side person”, and “gaze at the other”. Gaze events were manually annotated features defined as gazing at some object, that is, when the participant focused his/her visual attention on a particular object for a certain period of time (more than 200 ms). Figure 2 shows an example of annotation using ELAN. The annotators observed the videos and manually annotated each event.

Table 1 lists the basic statistics of the speaker’s and listeners’ gazing ratios in L1 conversations. The average of speaker’s gazing ratios, which shows how long the speaker gazed at other participants during her or his utterance, is 27.9%, and the average of listener’s gazing ratios, which shows how long each listener gazed at the speaker during the utterance, is 45.4%. Consequently, people gaze 1.6 times more while listening than while speaking in L1 conversations. This statistical result is highly consistent with the findings of Vertegaal et al. (2001) in multiparty conversations, who reported that people gaze 1.6 times more while listening than while speaking, even though there are differences in languages, conversation topics, and the number of participants between the two sets of multimodal data on multiparty conversations.

**Table 1:** Averages of speaker’s, listener’s, and Role-based Gazing Ratios in L1 conversations

Gazing person–gazed person	Ratio in L1 conv. (%)
Speaker–listener	27.9
Current speaker–next speaker	38.4
Current speaker–other participant	19.3
Listener–speaker	45.4
Next speaker–current speaker	48.9
Other participant–current speaker	42.5

Table 1 also lists the averages of Role-based Gazing Ratios, which were introduced to analyze eye gaze from the perspective of conversational interaction (Ijuin et al. 2015). The roles of participants are classified into current speaker, next speaker, and the other participant (not next speaker) by considering the context of utterance sequences. The average of Role-based Gazing Ratios showing how long the speaker gazed at the next speaker during her or his utterance is 38.4% in L1 conversations, whereas this value for gazing at the other participant is 19.3%. On the other hand, there is a relatively small difference between the averages of listener’s gazing ratios for the next speaker and for the other participant, which are 48.9 and 42.5%, respectively. In other words, the speaker gazes more at the interlocutor who is to be the next speaker than at the other participant, whereas the two listeners gaze at the speaker to nearly the same degree. These observations seem to accord with the findings of Jokinen et al. (2013), who reported that the speaker exerts more influence than the other partners in coordinating interaction in general and, in particular, anticipates the next speaker by focusing her or his overt attention on this person.

These observations suggest that this study’s multimodal corpus has several common features of eye gaze with previous multimodal corpora and thus is suitable for comparing the differences in eye gaze between L1 and L2 conversations.

## Method

### Methodology of Analyzing Relation Between Eye Gaze and Floor Apportionment

Yamamoto et al. (2015) and Ijuin et al. (2015) reported that the quantity of listeners’ eye gazes toward other participants is significantly different between L1 and L2 conversations. Such a quantitative difference may be reflected as a difference in interaction structure, although various functions of eye gaze are expected to be common to both L1 and L2 conversations. In this research, we compare the eye gaze activities between L1 and L2 conversations quantitatively, focusing on the difference in eye gaze activities for floor apportionment between L1 and L2 conversations.

Table 2 shows an example of transcribed utterances and eye gazes during a single turn drawn from the multimodal corpus. Here, the gaze targets of either speaker or listeners change during the utterances. Analyses based on gazing ratios do not contain information concerning these eye gaze transition patterns, including mutual gaze. Horiuchi et al. (2017) classified these and developed several types of next speaker prediction models using the eye gaze transition patterns. They trained various parameters of support vector machines (SVMs) to predict the next speaker based on eye gaze transition patterns and conducted experiments to determine what features would be most efficient for predicting the next speaker. They also compared the accuracies of prediction models in both L1 and L2 conversations. Their experiment is summarized in “Appendix 1”. The result shows that there was no difference between a model based on gazing ratio and a model using gaze transition patterns including mutual gaze. Although the gazing ratio does not contain detailed information concerning these eye gaze transition patterns, gazing ratio provides almost equal accuracy as the model using eye gaze transition patterns to predict the next speaker in triad conversations. Based on this result, we used gazing ratio as a more tractable measure for comparing eye gaze activities between L1 and L2 conversations.

**Table 2:** Example of conversation flow and eye gazes during an utterance in a single turn in L2 conversation

No.	Speaker	Utterance	A’s gaze	B’s gaze	C’s gaze
1	A:	I think that (pause)	B	A	A
2	A:	Its (pause)	None	None	A
3	A:	Tool is not important but the person is important to bring the uninhabited island I think for example especially mother (pause)	B → C → B	A	A
4	A:	To cook (pause)	B	None → A	A
5	A:	Something (pause)	C	None → A	A
6	A:	Foods I think umm	B → none → B	A	A
7	B:	(Overlapped “umm”) In my opinion (pause)	A	A	A → B
8	B:	A doctor	B	A	B

None represents objects other than participants A, B, and C. Pause represents the interval between utterances with a speaker in which the duration is more than 500 ms

The participant roles are classified into three types: current speaker (CS) as the speaker of the utterance, next speaker (NS) as the participant who takes the floor after the current speaker releases the floor, and other participant (OP) who is not involved in floor apportionment at that time. The average of Role-based Gazing Ratios is defined as

1 $${\text{Average of Role-based Gazing Ratios }}\left( {\text{Gazing Ratio}} \right) = \frac{1}{n}\mathop \sum \limits_{i = 1}^{n} \frac{{DG_{{jk_{\left( i \right)} }} }}{{DSU_{\left( i \right)} }} \times 100\left( {\text{\% }} \right),$$

where *DSU*_(*i*)_ and *DG*_*jk*(*i*)_ represent the duration of the *i*-th utterance and the duration of participant *j* gazing at participant *k* during that utterance, respectively. Role-based Gazing Ratio is calculated for each group. In the following sections, Gazing Ratio is used as the shorthand notation of the average of Role-based Gazing Ratios.

To analyze quantitatively the relation between eye gaze and floor apportionment, we classified utterances into two groups: utterances after which the speaker held the floor and utterances after which the other takes the floor (referred to as utterances “with floor-hold” and utterances “with floor-switch”, respectively, in the following discussion). In order to compare the eye gazes of each participant role between the two conditions (utterances with floor-hold and with floor-switch), Gazing Ratio was calculated for each condition. In the following sections, Gazing Ratio calculated for the utterances with floor-hold is shortened to Gazing Ratio with floor-hold, and Gazing Ratio calculated for the utterances with floor-switch is termed Gazing Ratio with floor-switch.

**Fig. 3:**
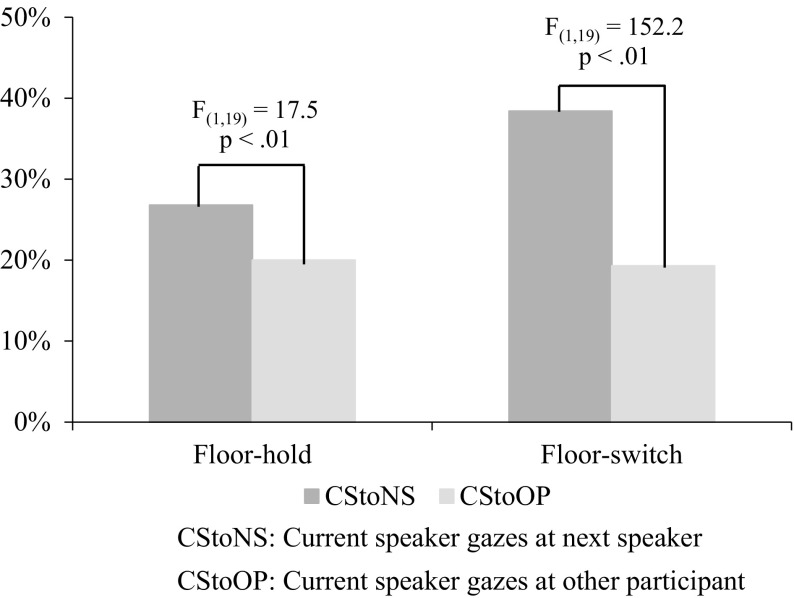
Averages of Gazing Ratios for the current speaker in utterances with floor-hold and those with floor-switch in L1 conversations

Figure 3 shows Gazing Ratios with floor-hold and with floor-switch for the current speaker gazing to the next speaker (CStoNS) and to the other participant (CStoOP) in L1 conversations; the *F* values and *p* values above the graph show a significant difference revealed by an ANOVA test as argued in the following analysis. Both Gazing Ratios with floor-hold and with floor-switch for CStoNS are larger than those for CStoOP. The difference between Gazing Ratios with floor-switch for CStoNS and for CStoOP is 19.1%, whereas the difference between Gazing Ratios with floor-hold for these gaze pairs is only 6.8%. These results show that the speaker gazes more at the next speaker than at the other participant in utterances for floor apportionment in triad conversations. Furthermore, these results accord with the findings on mutual gaze that describe the short moment of gazing at the point of turn transition, when both the current speaker and the next speaker look at each other (Argyle and Dean 1965; Kendon 1967), as well as with the findings of Jokinen et al. (2013) in which the speaker has the main role in anticipating the next speaker, and especially their suggestion that this selection is performed by eye gaze and focusing one’s visual attention on the partner who is likely to be the next speaker.

The difference in L1 conversations between Gazing Ratios with floor-hold and with floor-switch for CStoNS is 11.6%, whereas the difference between gaze conditions for CStoOP is less than 1%. This result shows that the current speaker gazes more at the next speaker in utterances with floor-switch than with floor-hold, although the current speaker gazes at the other participant in utterances with floor-switch to the same degree as in utterances with floor-hold.

These results suggest that the comparison between Gazing Ratios with floor-hold and with floor-switch for CStoNS is expected to serve as a suitable quantitative measure to analyze the efficiency of eye gaze for coordinating floor apportionment in conversations. In the following, we compare Gazing Ratios for CStoNS from two viewpoints, between language differences and between floor apportionment, to compare the functionality of eye gaze for coordinating floor apportionment in L1 and L2 conversations.

Table 3 compares the proportions of utterances with floor-hold and utterances with floor-switch in both languages and conversation topics. The proportions of utterances with floor-switch are larger than those with floor-hold in both languages, and the difference between them is much larger in L1 conversations than in L2 conversations. No difference between conversational topics was found in either L1 or L2 conversations.

**Table 3:** Proportions of utterances with floor-hold and with floor-switch for each conversation topic and language

	L1 conv.		L2 conv.	
	Free-flowing (%)	Free-flowing (%)	Goal-oriented (%)	Goal-oriented (%)
Floor-hold	22.2	38.7	41.8	22.6
Floor-switch	77.8	61.3	58.2	77.4

### Analyses of Eye Gaze from Perspective of Floor Apportionment

We formulated two hypotheses for answering the two questions posed in the Introduction: (1) the speaker’s eye gaze affects floor apportionment more significantly in L2 conversations than in L1 conversations, (2) longer listeners’ eye gazes in L2 conversations function efficiently in smooth floor apportionment. In order to test these hypotheses on the speaker’s and listeners’ eye gazes in L2 conversations, we conducted an ANOVA test to investigate the statistical differences between languages, conversation topics, gaze channels (gazer-target pairs), and floor apportionment conditions (floor-hold or floor-switch). The detailed results of the ANOVA test are given in “Appendix 2”, and the excerpted results directly related to the hypotheses are presented in this section.

### Analyses of Speaker’s Eye Gaze

**Fig. 4:**
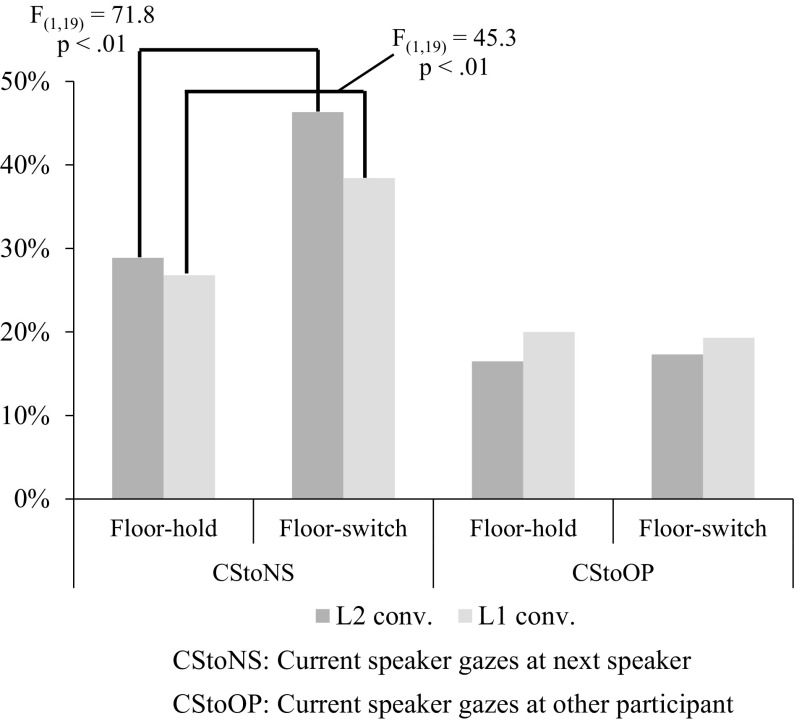
Averages of Role-based Gazing Ratios for the current speaker gazing to the next speaker (CStoNS) and to the other participant (CStoOP) in utterances with floor-hold and with floor-switch in L1 and L2 conversations

To test the first hypothesis, we calculated Gazing Ratios for CStoNS (the eye gaze of the current speaker toward the next speaker) in both conditions of floor apportionment and in both languages. Figure 4 compares these Gazing Ratios and also shows the results of Gazing Ratios for CStoOP for reference. As the figure shows, the current speaker gazes more at the next speaker than at the other participant in both conditions of floor apportionment in L2 conversations as well as in L1 conversations. There is a greater difference between Gazing Ratios with floor apportionment for CStoNS in L2 than in L1 conversations. Table 4 gives a summary of Gazing Ratios with floor-hold and with floor-switch and also shows the differences between the two. The rightmost column shows the differences between Gazing Ratios with floor-hold and with floor-switch. The difference in Gazing Ratios with floor-switch between CStoNS and CStoOP (the eye gaze of the current speaker toward the other participant) in L2 conversations is much larger than that in L1 conversations. These observations show that the speakers gaze more at a listener who will be the next speaker in utterances with floor-switch in L2 conversations than in L1 conversations.

**Table 4:** List of averages of Role-based Gazing Ratios with floor-hold and with floor-switch for current speaker in L1 and L2 conversations

	CStoNS (%)	CStoOP (%)	Diff. (%)
Floor-hold in L1	26.8	20.0	6.8
Floor-switch in L1	38.4	19.3	19.1
Floor-hold in L2	28.8	16.5	12.3
Floor-switch in L2	46.3	17.3	29.0

### Analyses of Listeners’ Eye Gazes

**Fig. 5:**
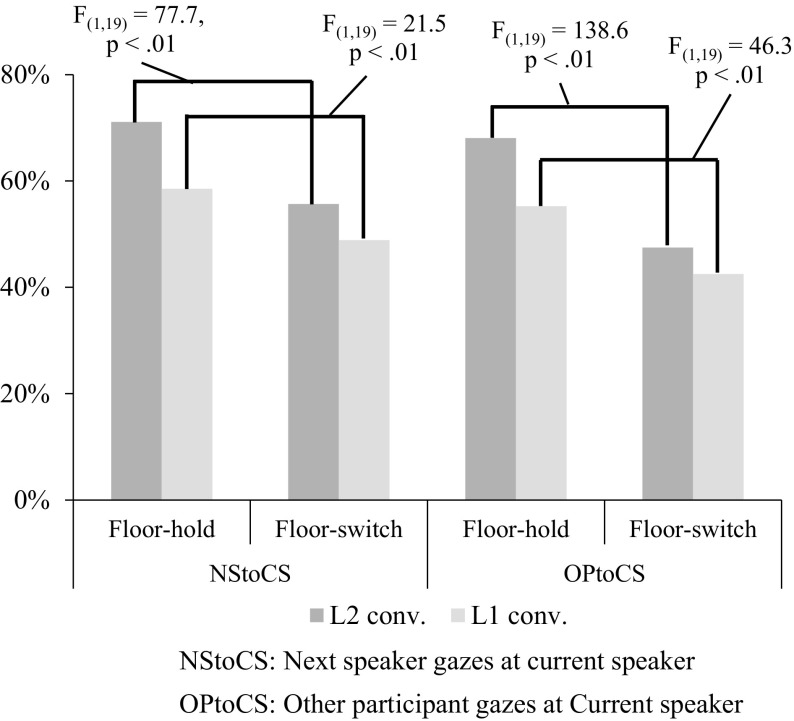
Averages of Role-based Gazing Ratios for the next speaker gazing to the current speaker (NStoCS) and for the other participant gazing to the current speaker (OPtoCS) in utterances with floor-hold and with floor-switch in L1 and L2 conversations

We calculated Gazing Ratios for both listeners’ gaze toward the speaker. Figure 5 shows Gazing Ratios with floor-hold and with floor-switch for NStoCS (eye gaze of the next speaker toward the current speaker) and OPtoCS (eye gaze of the other participant toward the current speaker). As shown in this figure, Gazing Ratios with floor-switch for both NStoCS and OPtoCS are smaller than Gazing Ratios with floor-hold for those gaze pairs. This result shows that both the next speaker and the other participant gaze at the current speaker significantly less in utterances with floor-switch than in utterances with floor-hold. The result also shows that those differences of floor apportionment are larger in L2 conversations than in L1 conversations.

These results demonstrate that the listeners gaze less at the speaker in utterances with floor-switch than in utterances with floor-hold in both L1 and L2 conversations. This suggests the possibility that both listeners mainly gaze at the speaker while he/she holds the floor and they somehow notice the difference between utterances with floor-hold and with floor-switch.

## Discussion

To clarify the differences in speaker’s eye gaze for floor apportionment between L1 and L2 conversations, we conducted a quantitative comparison of speaker’s eye gaze in utterances with floor-switch and those with floor-hold in both languages.

The analyses of the speaker’s eye gaze in the previous section revealed the following observations:

1. Concerning the speaker’s eye gaze target, the speaker gazes more at the next speaker than at the other participant in both utterances with floor-switch and those with floor-hold in L2 conversations as well as in L1 conversations.
2. Concerning the difference between the two conditions of floor apportionment, the speaker gazes more at the next speaker in utterances with floor-switch than in utterances with floor-hold in both L1 and L2 conversations.
3. Concerning the difference between languages, the speaker gazes more at the next speaker in utterances with floor-switch, in comparison with utterances with floor-hold, in L2 than in L1 conversations.

Observations (1) and (2) imply that the speaker directed his/her gaze to who will be the next speaker in both L1 and L2 conversations, and the speaker gazed more at the recipient during the utterance with floor-switch in almost the same practice adopted in L1 conversations. Observation (3) implies the probability that the gazed-at participant will be the next speaker is higher in L2 conversations than in L1 conversations. This shows that the speaker more likely focuses his/her overt attention to the next speaker in L2 conversations than in L1 conversations.

These observations suggest that the functionality of speaker’s eye gaze in floor apportionment is likely to be common to L1 and L2 conversations; however, the probability that the gazed-at participant will be the next speaker is higher in L2 conversations than in L1 conversations. This further suggests that the speaker’s eye gaze affects floor apportionment more significantly in L2 conversations than in L1 conversations, which supports our first hypothesis. Lower conversational competence in L2 might reinforce this interpretation because the lack of vocabulary, shortage of grammatical knowledge, and poor prosodic control may hinder L2 interlocutors in sending verbal signals for floor apportionment. The speaker’s directing gaze does not automatically ensure who will actually speak next because additional interactive contingencies can intervene, but the probability that the gazed-at participant, who perceives the speaker’s eye gaze, will be the next speaker is higher in L2 conversations than in L1 conversations.

As Lerner (2003) pointed out, to understand that he/she is the intended recipient, the gazed-at participant must see the gaze, and others may also need to see this to comprehend that someone else has been indicated as the intended recipient. In L2 conversations in which the speaker proactively directs his/her gaze to the intended recipient, it is expected that the listeners need to direct their gaze to the speaker in order not to miss the visual signals of floor apportionment given by the speaker. There is a possibility that the signal of floor apportionment from the speaker might be shown by his/her eye gaze more explicitly in L2 conversations than in L1 conversations; at least the statistical results show that Gazing Ratio for the speaker toward the next speaker is larger in L2 conversations than in L1 conversations.

The analysis results of listeners’ eye gazes in the previous section demonstrated the following observations:

4. Concerning the difference between the two conditions of floor apportionment, both the next speaker and the other participant gaze less at the speaker in utterances with floor-switch than in those with floor-hold in both L1 and L2 conversations.
5. Concerning the difference between languages, the differences of Gazing Ratios for NStoCS between utterances with floor-hold and with floor-switch are larger in L2 than in L1 conversations.

The observation of the listeners’ eye gaze (4) suggests the possibility that the listeners notice the utterance with floor-switch and decrease their gaze attention to the speaker after noticing the point of floor switch. This result is consistent with Kendon (1967) that the next speaker reduces the amount of gaze at the current speaker in order to prepare the utterance in dyadic conversations. This suggests that the next speakers need more concentration to prepare for the next utterance and to reduce the visual attention to the speaker due to the lack of L2 proficiency. The other observation (5) shows that the decrease in the next speaker’s eye gaze is larger in L2 than in L1 conversations. This larger decrease may lead to the higher chance that the speaker notices this change more easily in L2 conversations than in L1 conversations.

Although we may not yet be able to prove our second hypothesis to a sufficient level, our experimental results seem to support the hypothesis that longer listeners’ eye gazes in L2 conversations function efficiently in smooth floor apportionment. That is to say, the speakers need to make use of visual signals for floor apportionment by using eye gaze to compensate for their lower proficiency in L2. Meanwhile, the listeners need to gaze at the speaker in order not to miss the visual signals of floor apportionment given by the speaker, regardless of whether the listeners are gazed at by him/her.

## Conclusion and Future Work

We compared eye gazes of interlocutors in utterances for floor apportionment in triad conversations in L2 with those in L1 and obtained five key findings:

1. Concerning the speaker’s eye gaze target, the speaker gazes more at the next speaker than at the other participant in both utterances with floor-switch and with floor-hold in L2 conversations as well as in L1 conversations.
2. Concerning the speaker’s eye gaze difference between the two conditions of floor apportionment, the speaker gazes more at the next speaker in utterances with floor-switch than in utterances with floor-hold in both L1 and L2 conversations.
3. Concerning the speaker’s eye gaze difference between languages, the speaker gazes more at the next speaker in utterances with floor-switch, in comparison with utterances with floor-hold, in L2 conversations than in L1 conversations.
4. Concerning the listeners’ eye gaze difference between the two conditions of floor apportionment, both the next speaker and the other participant gaze less at the speaker in utterances with floor-switch than with floor-hold in both L1 and L2 conversations.
5. Concerning the next speaker’s eye gaze difference between languages, the differences of Gazing Ratio for the next speaker’s eye gaze toward the speaker between utterances with floor-hold and with floor-switch are larger in L2 conversations than in L1 conversations.

These findings of the speaker’s eye gaze confirm our first hypothesis that the speaker’s eye gaze affects floor apportionment more significantly in L2 than in L1 conversations, since the gazed-at participant in utterances with floor-switch will be the next speaker at higher probability in L2 conversations than in L1 conversations. These findings of the listeners’ eye gazes are consistent with our second hypothesis that longer listeners’ eye gazes in L2 conversations function efficiently in smooth floor apportionment, although we may not yet to be able to prove our second hypothesis to a sufficient level. The larger decrease in the next speaker’s eye gaze in utterances with floor-switch compared with those with floor-hold may lead to the higher possibility that the speaker notices this change more easily in L2 than in L1 conversations.

We assume that the cause of the difference in eye gaze activities and their effects reported here may not be specific to the L1 versus L2 contrast but rather generalizable due to differences in linguistic proficiency. In order to explore this idea, we are planning to create a multi-modal corpus in a controlled way with groups categorized by L2 proficiency levels and with a balanced number of participants.

## Appendix 1: Analysis of Gaze Pattern and Flow of Conversation

Horiuchi et al. developed several types of next speaker prediction models using various eye gaze features during utterances with floor-switch and with floor-hold in order to examine which feature would be most efficient for predicting the next speaker. They compared the accuracies of these prediction models in both L1 and L2 conversations. In this appendix, we overview the Gaze Transition Model, Last Model, and Gazing Ratio Model as the three principal models.

The Gaze Transition Model uses the transition patterns of participants’ eye gazes, as shown in Table 2, and mutual gaze occurrences as the results of the transition of participants’ eye gazes during the utterance as features, and it ignores the duration of eye gaze. They enumerated the distribution of all gaze transition patterns during the utterance and listed those occurring in more than 1% of the entire distribution. The gaze patterns in less than 1% were classified as one group. The gaze transition of each participant was given to the prediction model as a feature vector. The feature vector was constructed with the listed gaze transition patterns as the corresponding dimensions. For example, if the specific gaze pattern was shown in the utterance, its dimension was 1 and other dimensions’ values were 0. They gave that vector for each participant to the prediction model.

The Last Model uses the gaze target information of each participant at the end of the utterance and also ignores the duration of eye gaze. The possible gaze targets of each participant are the left participant, the right participant, or not participant.

The Gazing Ratio Model uses the values of features calculated by the same equation of Gazing Ratio for each utterance to predict who will be the next speaker.

The results indicate that the accuracies in L2 conversations are higher with all models than those in L1 conversations as shown in Table 5. The results also show that there is no difference in accuracy among all three models. Furthermore, from these results, the information of transition patterns of the participants’ eye gazes did not provide any improvement of prediction accuracy of the next speaker in comparison with the information of the participant to whom the speaker gazed at the end of the utterance or to whom the speaker gazed the most during the utterance.

**Table 5:** Accuracies of each model in L1 and L2 conversations

	L1 conv. (%)	L2 conv. (%)
Gaze Transition Model	43.6	52.9
Last Model	44.2	53.0
Gazing Ratio Model	43.4	53.9

## Appendix 2: Results of ANOVA Test

In order to analyze the eye gaze difference of languages and floor apportionment, an ANOVA test was conducted with language (L1 or L2), conversation topic (free-flowing or goal-oriented), gaze channels (gazer-target pairs), and floor apportionment (floor-hold or floor-switch) being within-subject factors. The *F* value and *p* value in each condition are shown in Table 6 (note that the results are numbered for reference in the explanations below). There was neither a significant main effect of conversation topic nor a significant interaction involving it, so we focus on the language, gaze channel, and floor apportionment factors in the following.

**Table 6:** Excerpt results related to our analyses of ANOVA tests with languages, gaze channel, and floor apportionment difference as within-subject factors

		*F* value	*p* value
2nd Order interactions			
(1)	Interaction of floor apportionment × language × gaze channel	*F*_(5,95)_ = 3.3	0.01
(2)	Simple interaction: floor apportionment × gaze channel		
(2.1)	L1 conversations	*F*_(5,95)_ = 38.7	0.00
(2.2)	L2 conversations	*F*_(5,95)_ = 98.0	0.00
(3)	Simple interaction: language × gaze channel		
(3.1)	Floor-hold	*F*_(5,95)_ = 17.2	0.00
(3.2)	Floor-switch	*F*_(5,95)_ = 20.2	0.00
(4)	Simple interaction: floor apportionment × language		
(4.1)	CStoNS	*F*_(1,19)_ = 7.9	0.01
(4.2)	NStoCS	*F*_(1,19)_ = 4.3	0.05
(4.3)	OPtoCS	*F*_(1,19)_ = 11.1	0.00
(5)	Simple-simple main effect: floor apportionment		
(5.1)	CStoNS × L1 conversation	*F*_(1,19)_ = 45.3	0.00
(5.2)	CStoNS × L2 conversation	*F*_(1,19)_ = 71.8	0.00
(5.3)	NStoCS × L1 conversation	*F*_(1,19)_ = 21.5	0.00
(5.4)	NStoCS × L2 conversation	*F*_(1,19)_ = 77.7	0.00
(5.5)	OPtoCS × L1 conversation	*F*_(1,19)_ = 46.3	0.00
(5.6)	OPtoCS × L2 conversation	*F*_(1,19)_ = 138.6	0.00
(6)	Simple-simple main effect: language		
(6.1)	CStoNS × floor-hold	*F*_(1,19)_ = 1.1	0.31
(6.2)	CStoNS × floor-switch	*F*_(1,19)_ = 33.4	0.00
(6.3)	NStoCS × floor-hold	*F*_(1,19)_ = 27.1	0.00
(6.4)	NStoCS × floor-switch	*F*_(1,19)_ = 17.5	0.00
(6.5)	OPtoCS × floor-hold	*F*_(1,19)_ = 28.1	0.00
(6.6)	OPtoCS × floor-switch	*F*_(1,19)_ = 18.1	0.00

The results of the ANOVA test show a significant interaction of floor apportionment, language, and gaze channel difference [result (1) in Table 6]. The sub-effect test shows significant simple interactions of floor apportionment and gaze channel in both L1 and L2 conversations [L1 conversations: result (2.1), L2 conversations: result (2.2)]. The results show significant simple interactions of language and gaze channel in both with floor-hold and with floor-switch [floor-hold: result (3.1), floor-switch: result (3.2)]. The results also show significant simple interactions of language and floor apportionment in CStoNS and OPtoCS [CStoNS: result (4.1), OPtoCS: result (4.3)] and marginally significant simple interactions of language and floor apportionment in NStoCS [result (4.2)].

We conducted tests for simple–simple main effects (simple–simple means specifying two factors) of the floor apportionment in each gaze channel. The results show that there are significant simple–simple main effects of floor apportionment difference in CStoNS in both L1 and L2 conversations [L1 conversations: result (5.1), L2 conversations: result (5.2)]. The results for listeners’ eye gazes toward the speaker also show significant simple–simple main effects of apportionment difference in both L1 and L2 conversations [results from (5.3) to (5.6)].

We analyzed the language difference in each gaze channel. The results show that there is a significant simple–simple main effect of language difference in CStoNS with floor-switch [result (6.2)], whereas there was no significant simple–simple main effect of language difference in CStoNS with floor-hold [result (6.1)]. For listeners’ eye gazes, there are significant simple–simple main effects of language difference in both NStoCS and OPtoNS in both conditions of floor apportionment [results from (6.3) to (6.6)].

Analysis results that show the significant differences related to our hypotheses [results from (5.1) to (5.4)] were described in Sect. 3.
